# Linking PANSS negative symptom scores with the Clinical Global Impressions Scale: understanding negative symptom scores in schizophrenia

**DOI:** 10.1038/s41386-019-0363-2

**Published:** 2019-03-05

**Authors:** Stefan Leucht, Ágota Barabássy, István Laszlovszky, Balázs Szatmári, Károly Acsai, Erzsébet Szalai, Judit Harsányi, Willie Earley, György Németh

**Affiliations:** 1 Department of Psychiatry and Psychotherapy, Technische Universität München, Munich, Germany; 2 Medical Division, Gedeon Richter Plc, Budapest, Hungary; 3 Clinical Development, Allergan plc, Madison, NJ USA

**Keywords:** Schizophrenia, Medical research

## Abstract

Understanding how rating scale improvement corresponds to a clinical impression in patients with negative symptoms of schizophrenia may help define the clinical relevance of change in this patient population. We conducted post hoc equipercentile linking analyses of Positive and Negative Syndrome Scale (PANSS) outcomes (e.g., PANSS-Factor Score for Negative Symptoms [FSNS]) with Clinical Global Impressions-Improvement (CGI-I) and -Severity (CGI-S) ratings on data from patients treated with cariprazine (*n* = 227) or risperidone (*n* = 229) in a clinical study evaluating negative symptoms in schizophrenia. Patients were prospectively selected for persistent, predominant negative symptoms of schizophrenia (PNS), and minimal positive/depressive/extrapyramidal symptoms. Linking results demonstrated that greater improvement on PANSS-derived measures corresponded to clinical impressions of greater improvement, as measured by the CGI-I, and less severe disease states, as measured by the CGI-S. For example, CGI-S scores of 1 (normal), 2, 3, 4, 5, and 6 (severely ill) corresponded to PANSS-FSNS scores of 7, 13, 19, 24, 29, and 35, respectively. Likewise, CGI-I scores of minimally improved, much improved, and very much improved corresponded to a change from baseline in PANSS-FSNS scores of −27%, −49%, and −100%, respectively. These are important findings for the interpretation of the results of trials in patients with persistent negative symptoms.

## Introduction

Schizophrenia is a neuropsychiatric syndrome characterized by positive (i.e., hallucinations, delusions) and negative (i.e., blunted affect, anhedonia) symptoms, which reflect what is seen in the clinic and allow for categorization of symptoms into distinct symptom domains. Although positive symptoms are amenable to treatment with antipsychotic medication, negative symptoms are responsible for much long-term morbidity and functional impairment in patients with schizophrenia; [1] in point of fact, functioning and improvement in functioning have been shown to be more strongly correlated with negative symptom factors than with positive and other symptom factors [2]. Unfortunately, finding effective treatment for negative symptoms has proven to be a challenge. Since most negative symptom improvement occurs in tandem with improvement in positive, depressive, or extrapyramidal symptoms (EPS), evaluating genuine treatment effect on negative symptoms requires a well-designed trial in a patient population prospectively selected for primary and persistent negative symptoms.

In drug research in schizophrenia, treatment effect is routinely determined by mean change in score on a symptom-based rating scale, such as the Positive and Negative Syndrome Scale (PANSS) [3]. The PANSS total score is widely used to assess schizophrenia psychopathology in clinical trials, with the thresholds for clinically meaningful change already established by cutoff scores for patients with acute exacerbation of schizophrenia [4]. However, defining what qualifies as clinically meaningful change on this, or any, rating scale is a methodological concern in trials evaluating negative symptoms [5]. By definition, change is clinically meaningful only if it improves patient functioning and quality of life. Since most trials that assess negative symptoms also include patients with positive symptoms, negative symptom improvement that occurs secondarily to changes in positive or depressive symptoms is prone to ambiguous interpretation [6]. Understanding negative symptoms that improve independently of improvement in other symptom domains and represent genuine treatment effect could help advance an understanding of what constitutes clinically meaningful change in patients with schizophrenia.

Cariprazine, a dopamine D_3_-preferring D_3_/D_2_ receptor partial agonist and serotonin 5-HT_1A_ receptor partial agonist, is approved in Europe and the United States for the treatment of adults with schizophrenia. In a prospective clinical trial in patients with stable schizophrenia and persistent, predominant negative symptoms (PNS) (EudraCT 2012-005485-36), the difference in negative symptom improvement was statistically significant in favor of cariprazine versus the active-comparator risperidone [7]. The primary outcome measure was change from baseline to week 26 in the PANSS-Factor Score for Negative Symptoms (PANSS-FSNS) [8], a PANSS-derived scale that is also referred to in the literature as the Marder Factor for Negative Symptoms. Patients were specifically selected for PNS in schizophrenia, with no notable positive symptoms, depressive symptoms, or EPS present at baseline. This ensured that changes observed in negative symptoms were genuine treatment effect and did not occur secondarily to changes in these other symptom domains (i.e., pseudospecifically). Of note during the trial, changes in positive and depressive symptoms, and EPS, were small and similar for cariprazine and risperidone, further supporting the observation of an actual treatment effect for cariprazine. As such, this prospectively defined cariprazine trial provided us with a unique opportunity to evaluate the clinical relevance of negative symptom change that was not biased by the usual confounding factors in most negative symptom trials.

The Clinical Global Impressions (CGI) Scale [9], which was an additional outcome measure in the cariprazine negative symptom trial, is a short rating scale that was designed to introduce clinical meaning into drug trials by describing a patient’s overall clinical state as a global impression. Since little is known about how change in PANSS negative symptom outcomes correspond to clinically judged CGI severity and improvement during treatment, anchoring a PANSS negative scale to the CGI allows nominal translation of data across the scales and gives us the ability to clinically quantify negative symptom change.

Using equipercentile linking [10], a well-established statistical method for linking rating scales [11], we conducted post hoc analyses to determine what points on the PANSS corresponded to specific CGI ratings so we could assess the clinical meaning of negative symptom changes in the unique PNS patient population from the cariprazine trial. These linking analyses could help advance our understanding of negative symptoms and improve the interpretation of clinical trial results for patients with schizophrenia.

## Methods

The cariprazine negative symptom study was a randomized, double-blind, active-controlled study conducted in 11 European countries in patients with schizophrenia and PNS; detailed methods have been published previously (EudraCT number 2012-005485-36) [7]. The primary study was conducted in accordance with good clinical practice guidelines and the principles of the International Conference on Harmonisation; all patients provided written informed consent. In brief, the study consisted of a 4-week prospective lead-in period, a 26-week double-blind treatment period (2-week up-titration and 24-week continuation treatment), and a 2-week safety follow-up period. Patients were randomized (1:1) to once-daily cariprazine or risperidone; cariprazine 4.5 mg/d or risperidone 4 mg/d were the target doses.

Male and female patients (18–65 years of age, inclusive) had a diagnosis of schizophrenia according to *Diagnostic and Statistical Manual of Mental Disorders* (DSM-IV-TR) [12] criteria, with an onset of illness ≥2 years and a stable condition for at least 6 months. Clinical inclusion criteria required patients to have PNS for ≥6 months, a PANSS-FSNS score ≥24, and a score ≥4 on at least 2 of 3 PANSS negative symptom items (blunted affect [N1], passive/apathetic social withdrawal [N4], and lack of spontaneity and flow of conversation [N6]). Several exclusion criteria were applied, the most critical of which ensured that improvements in negative symptoms were not secondary to improvements in other psychopathological domains. Per these key criteria, patients were excluded for (1) positive symptoms defined as a score ≥4 on at least two of the positive PANSS items of delusions, hallucinatory behavior, grandiosity, suspiciousness, or unusual thought content; (2) moderate/severe depressive symptoms defined as a Calgary Depression Scale for Schizophrenia [CDSS] [13] total score >6; and 3) clinically relevant parkinsonism judged by the investigator or a score >3 on the sum of the first eight items of the Simpson-Angus Scale (SAS) [14].

### Rating scale measures for linking analysis

The PANSS-FSNS consists of 7 items: blunted affect (N1), emotional withdrawal (N2), poor rapport (N3), passive/apathetic social withdrawal (N4), lack of spontaneity/flow of conversation (N6), motor retardation (G7), and active social avoidance (G16). In addition to items N1, N2, N3, N4, and N6, the PANSS negative symptom subscale (PANSS-NSS) includes items N5 (difficulty in abstract thinking) and N7 (stereotyped thinking), and no general items. PANSS items are rated on a 7-point scale (1=absent, 2=minimal, 3=mild, 4=moderate, 5=moderate severe, 6=severe, and 7=extreme); because the absence of symptoms is equal to 1 point, the lowest possible total score on both PANSS scales is 7. To provide clinical meaning to PANSS-derived outcomes from the negative symptom trial, changes on these scales were linked to the 2 companion scales of the CGI: CGI-Severity (CGI-S) and CGI-Improvement (CGI-I) **(**Table 1). Given that patients admitted to the study had PNS and very limited positive symptoms, CGI scores most specifically reflected observed improvements and changes in severity of negative symptoms of schizophrenia.

**Table 1:** CGI subscales and scoring

Subscale	Score	Clinical description of score
Severity of Illness (CGI-S): considering your total clinical experience with this particular population, how mentally ill is the patient at this time?	1	Normal
	2	Borderline ill
	3	Mildly ill
	4	Moderately ill
	5	Markedly ill
	6	Severely ill
	7	Among the most extremely ill patients
Improvement (CGI-I): compared to the patient’s condition at admission to the project (prior to medication initiation), how much has this patient’s condition changed?	1	Very much improved
	2	Much improved
	3	Minimally improved
	4	No change from baseline
	5	Minimally worse
	6	Much worse
	7	Very much worse

*CGI-I* Clinical Global Impressions-Improvement, *CGI-S* Clinical Global Impressions-Severity

### Equipercentile linking analysis

Data from patients in the cariprazine- and risperidone-treatment arms were pooled for analyses; all patients with ≥1 postbaseline visit (modified intent-to-treat [ITT] population) were included in the analyses, and no missing values were imputed. Equipercentile linking was used to examine corresponding scores on the CGI-I and CGI-S, and the PANSS-FSNS and PANSS-NSS. The empirical percentile rank functions were calculated from the observed score distributions for the parameters to be equated; score values with the same percentile ranks were determined and plotted against each other using SAS program EQUIPERCENTILE [15]. To demonstrate the feasibility of equating the different scales, Spearman correlation coefficients were calculated to test the association between CGI and PANSS scores for the population at baseline and weeks 1, 2, 3, 4, 6, 10, 14, 18, 22, and 26; the total number of observations were also pooled across timepoints. Since the lowest PANSS-FSNS and -NSS subscale scores are 7, meaningful calculation of percentage change requires that 7 points be subtracted from PANSS-FSNS and PANSS-NSS values (BSL −7) as recommended in Obermeier et al. [16]. This adjustment was made for each percentage change analysis presented (percentage change results calculated without this adjustment are presented in Figure S1, S2). All statistical procedures were carried out using SAS 9.2.

To determine how the CGI-I and CGI-S corresponded to the PANSS-FSNS and PANSS-NSS, the following linking analyses were performed on data pooled by visit and treatment group using an observed cases approach: (1) CGI-S score with PANSS-FSNS/-NSS score; (2) CGI-I score with percentage change (BSL −7) on the PANSS-FSNS/-NSS; (3) CGI-I score with PANSS-FSNS/ -NSS score change; (4) CGI-S score change with PANSS-FSNS/-NSS score change; and (5) CGI-S score change with percentage change from baseline (BSL −7) on the PANSS-FSNS/-NSS. These same linking analyses were also conducted using by-weekly data to evaluate change over time and in subgroups of patients with PANSS-FSNS/-NSS baseline values at median or below (lower illness severity) and greater than median (higher illness severity) to evaluate the effect of baseline severity of illness.

## Results

### Patients

Analyses were conducted on data from 227 cariprazine-treated and 229 risperidone-treated patients who were included in the modified ITT population of the primary study. The mean age was ~40 years and more than half of the patients in each treatment group were men. The mean time since diagnosis of schizophrenia was between 11 and 12 years, and the majority of patients had <5 acute exacerbations of illness. Mean baseline PANSS-FSNS scores indicated the presence of negative symptoms of at least moderate severity (cariprazine=27.7; risperidone=27.5) [7].

### Correlation between the CGI and PANSS-FSNS/PANSS-NSS

Correlations between the PANSS and CGI for pooled observations during the 26-week study were statistically significant for all correlations (range, 0.534–0.762; *P* < 0.0001) across visits. Spearman correlation coefficients at every time point evaluated and across all variables analyzed are presented in Table 2. Correlations were smaller in weeks 1 and 2 when changes on the PANSS and CGI were still small and not necessarily in sync; the size of the correlations increased as the study progressed and changes on the scales corresponded.

**Table 2:** Spearman Correlation Coefficients: PANSS-FSNS, PANSS-NSS, and CGI Scores (observed cases)

Comparison	Baseline *n* = 456	Week 1 *n* = 456	Week 2 *n* = 451	Week 3 *n* = 443	Week 4 *n* = 436	Week 6 *n* = 426	Week 10 *n* = 406	Week 14 *n* = 383	Week 18 *n* = 373	Week 22 *n* = 362	Week 26 *n* = 343	Pooled^a^
CGI-S score vs PANSS-FSNS score	0.411	0.452	0.389	0.452	0.499	0.485	0.508	0.514	0.568	0.586	0.587	0.587
CGI-S score vs PANSS-NSS score	0.274	0.299	0.273	0.385	0.428	0.427	0.463	0.488	0.515	0.556	0.570	0.534
CGI-S score change vs PANSS-FSNS change	–^b^	0.185	0.349	0.540	0.590	0.588	0.549	0.592	0.627	0.618	0.623	0.668
CGI-S score change vs PANSS-NSS score change	–	0.202	0.375	0.520	0.599	0.591	0.548	0.572	0.590	0.621	0.622	0.663
CGI-I score vs PANSS-FSNS score change	–	0.373	0.530	0.659	0.679	0.655	0.658	0.683	0.718	0.682	0.686	0.758
CGI-I score vs PANSS-NSS score change	–	0.384	0.578	0.674	0.690	0.663	0.661	0.647	0.670	0.672	0.654	0.753
CGI-S score change vs PANSS-FSNS % change (BSL −7 points)	–	0.182	0.345	0.534	0.581	0.589	0.545	0.583	0.613	0.609	0.614	0.666
CGI-S score change vs PANSS-NSS % change (BSL −7 points)	–	0.195	0.370	0.526	0.604	0.600	0.571	0.587	0.602	0.633	0.630	0.671
CGI-I change vs PANSS-FSNS % change (BSL −7 points)	–	0.368	0.530	0.657	0.680	0.662	0.659	0.674	0.709	0.679	0.685	0.758
CGI-I change vs PANSS-NSS % change (BSL −7 points)	–	0.391	0.581	0.675	0.696	0.671	0.676	0.665	0.685	0.685	0.670	0.762

*P* < 0.0001 for all correlations.

*n* is the number of patients with data on both scales at each analysis point.

*BSL -7 points* baseline score minus 7 points, *CGI-I* Clinical Global Impressions-Improvement, *CGI-S* Clinical Global Impressions-Severity, *OC* observed cases, *PANSS-FSNS* Positive and Negative Syndrome Scale-Factor Score for Negative Symptoms, *PANSS-NSS* PANSS Negative Subscale Score

^a^The total number of pooled observations across visits for parameters with no baseline value (ie, change from baseline parameters or CGI-I) is 4079; the total number of pooled observations across visits for parameters with values that can evaluated at baseline is 4535

^b^There are no baseline values for score changes or CGI-I scores

### Linking CGI-S score with PANSS-FSNS and PANSS-NSS score

**Fig. 1:**
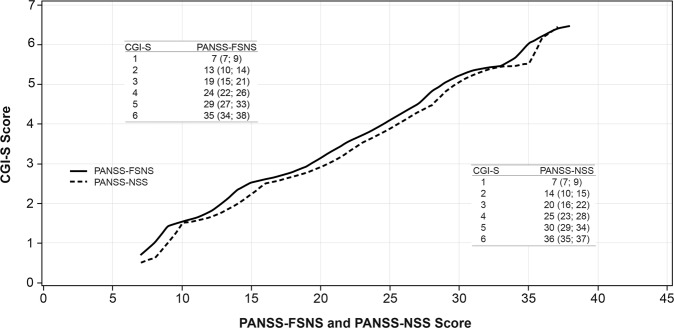
Linking CGI-S with PANSS-FSNS and -NSS score (pooled observations, observed cases). In the embedded tables, the nonparenthetical PANSS value is the average (best estimation) PANSS score that corresponds to a CGI-S score as a result of the equipercentile linking procedure; these CGI-PANSS pairs can be seen as the intersections in the figure. In parentheses, the full range of PANSS scores associated with the actual CGI-S score is presented. *CGI-S* Clinical Global Impressions-Severity, *FSNS* Factor Score for Negative Symptoms, *NSS* Negative Symptom subscale, *PANSS* Positive and Negative Syndrome Scale

This analysis investigated what scores on the PANSS-FSNS/-NSS linked to each CGI severity stage. When observations were pooled by visit and treatment group, CGI-S scores of 1 (normal), 2 (borderline mentally ill), 3 (mildly ill), 4 (moderately ill), 5 (markedly ill), and 6 (severely ill) corresponded to estimated PANSS-FSNS scores of 7, 13, 19, 24, 29, and 35, respectively **(**Fig. 1**)**. Similarly, CGI-S scores of 1, 2, 3, 4, 5, and 6 corresponded to estimated PANSS-NSS scores of 7, 14, 20, 25, 30, and 36.

### Linking CGI-I score with PANSS-FSNS and PANSS-NSS percentage change

**Fig. 2:**
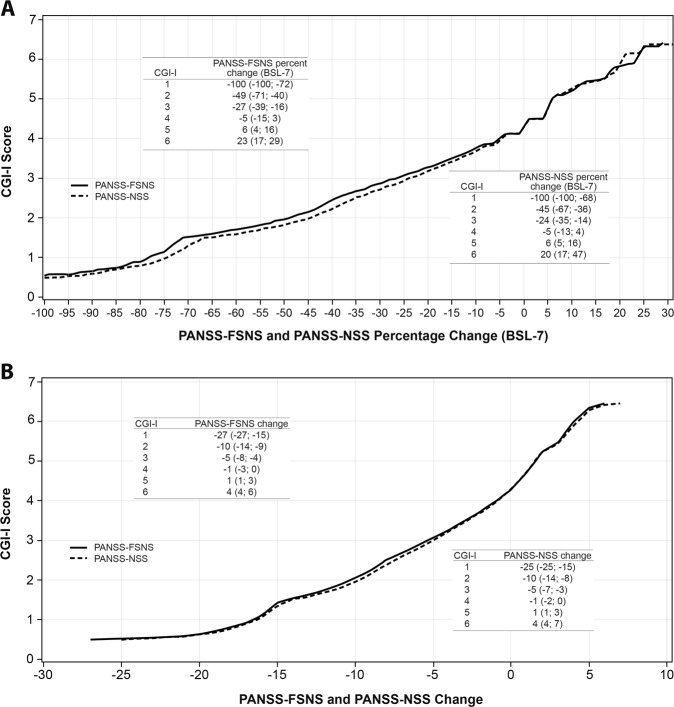
Linking CGI-I with PANSS-FSNS and -NSS (**a**) percentage change and (**b**) score change (pooled observations, observed cases). In the embedded tables, the nonparenthetical PANSS value is the average (best estimation) PANSS (**a**) percentage change or (**b**) score change that corresponds to a CGI-I score as a result of the equipercentile linking procedure; these CGI-PANSS pairs can be seen as the intersections in the respective figures. In parentheses, the full range of values for PANSS percentage or score change associated with the actual CGI-I score is presented. *BSL −7* baseline score minus 7 points, *CGI-I* Clinical Global Impressions-Improvement, *FSNS* Factor Score for Negative Symptoms, *NSS* Negative Symptom subscale, *PANSS* Positive and Negative Syndrome Scale

This analysis investigated what percentage of PANSS-FSNS/-NSS change from baseline is perceived as minimally improved, much improved, or very much improved from baseline on the CGI scale; in other words, for a patient to improve by a category on the CGI-I, how many PANSS-FSNS/-NSS percentage points of improvement are needed. A CGI-I score of 3 (minimally improved), 2 (much improved), and 1 (very much improved) corresponded to a change from baseline in estimated PANSS-FSNS scores of −27%, −49%, and −100%, respectively **(**Fig. 2a). CGI-I scores of minimally improved (3), much improved (2), and very much improved (1) corresponded to an estimated PANSS-NSS percentage change from baseline of −24%, −45%, and −100%, respectively.

### Linking CGI-I score with PANSS-FSNS and PANSS-NSS score change

This analysis investigated how much improvement in PANSS-FSNS/-NSS score was needed for a clinician to judge a patient as minimally improved, much improved, or very much improved on the CGI-I scale. Lower CGI-I scores, indicating greater improvement, corresponded to greater PANSS-FSNS score changes with a CGI-I score of 3 (minimally improved), 2 (much improved), and 1 (very much improved) corresponding to estimated PANSS-FSNS scores changes of −5, −10, and −27 (Fig. 2b). iSmilarly, CGI-I scores of 3, 2, and 1 corresponded to estimated PANSS-NSS scores of −5, −10, and −25.

### Linking CGI-S score change with PANSS-FSNS and PANSS-NSS score change

**Fig. 3:**
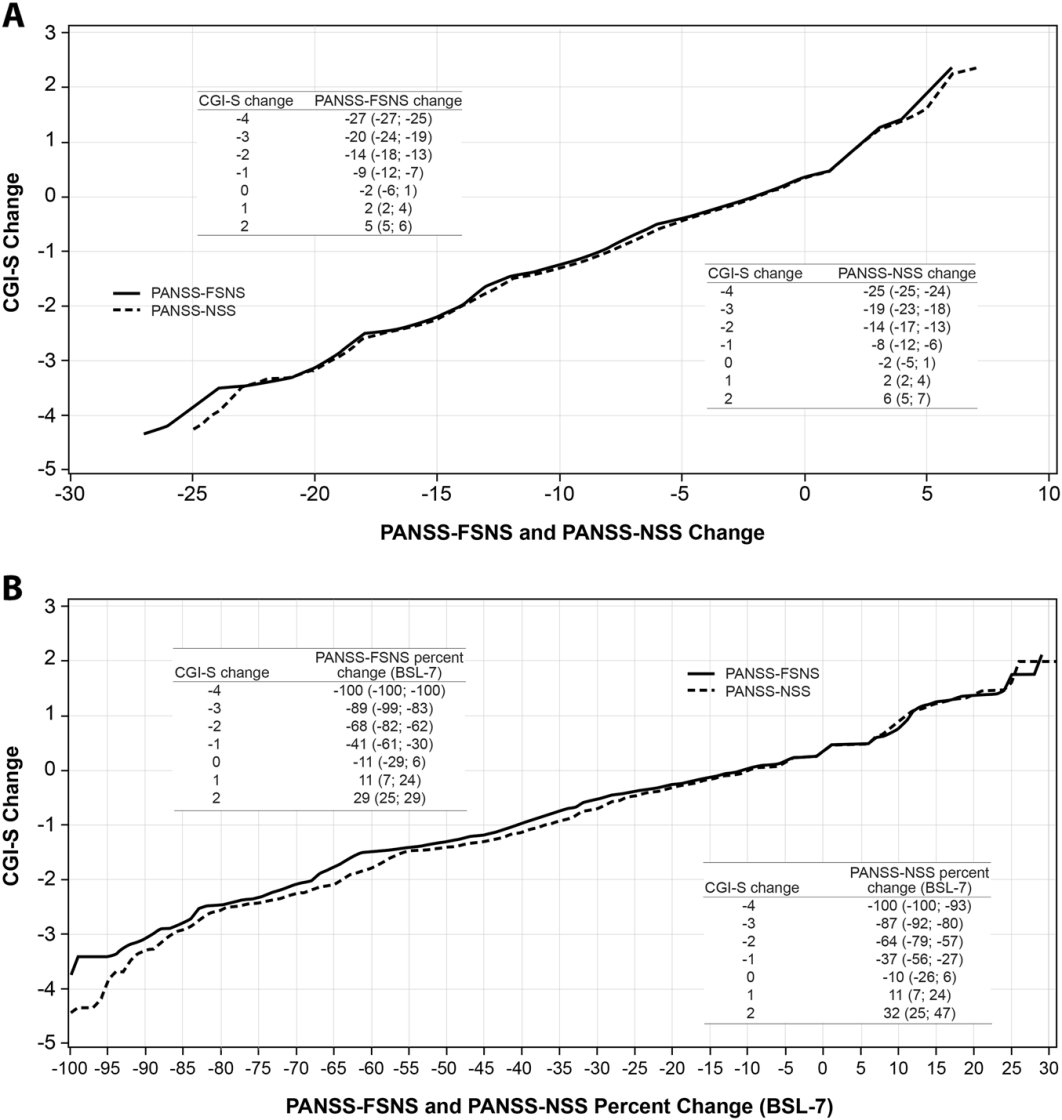
Linking CGI-S change from baseline with PANSS-FSNS and PANSS-NSS (**a**) change and (**b**) percentage change from baseline (pooled observations, observed cases). In the embedded tables, the nonparenthetical PANSS value is the average (best estimation) PANSS (**a**) score change or (**b**) percentage change value that corresponds to a CGI-S score change as a result of the equipercentile linking procedure; these CGI-PANSS pairs can be seen as the intersections in the respective figures. In parentheses, the full range of values for PANSS change associated with the actual CGI-S change is presented. *BSL −7* baseline score minus 7 points, *CGI-S* Clinical Global Impressions-Severity, *FSNS* Factor Score for Negative Symptoms, *NSS* Negative Symptom subscale, *PANSS* Positive and Negative Syndrome Scale

This analysis investigated the link between change from baseline in CGI-S score and change from baseline on PANSS-FSNS/-NSS; namely, how many PANSS-FSNS/-NSS points are necessary for a patient to switch into another CGI-S severity category. CGI-S absolute score changes of −1, −2, −3, and −4 were linked to estimated PANSS-FSNS improvement of −9, −14, −20, and −27, respectively **(**Fig. 3a**)**. On the PANSS-NSS, CGI-S absolute score changes of −1, −2, −3, and −4 were linked to estimated PANSS-NSS improvement of −8, −14, −19, and −25, respectively.

### Linking CGI-S score change with PANSS-FSNS and PANSS-NSS percentage change

This analysis investigated the link between CGI-S score change and PANSS-FSNS/-NSS percentage change to determine the percentage of PANSS-FSNS/-NSS change that is needed for a patient to switch into another CGI-S severity category. More CGI-S category changes, indicating decreased illness severity, were correlated with the greatest estimated percentage changes on the PANSS-FSNS: changes of 0, 1, 2, 3, and 4 steps were correlated to improvements of −11%, −41%, −68%, −89%, and −100%, respectively **(**Fig. 3b**)**. On the PANSS-NSS, a similar trend was observed: changes of 0, 1, 2, 3, and 4 steps were correlated to estimated improvements of −10%, −37%, −64%, −87%, and −100%, respectively. Of note, no change in CGI-S still corresponded to ~10% improvement on the adjusted PANSS-derived scales, suggesting that some PANSS improvement preceded a CGI-S category switch.

### Linking analysis depending on baseline severity of illness

The effect of severity of illness at baseline as shown by linking analyses in patients with baseline score ≤median (less severe illness) or >median (more severe illness) did not appear to be an important determinant of the amount of PANSS-FSNS or PANSS-NSS change in these analyses of patients with PNS **(**Figure S3, S4**)**.

### By-week linking analysis

When CGI-S and PANSS-FSNS were linked at baseline, a CGI-S score of 3 (mildly ill), 4 (moderately ill), 5 (markedly ill), and 6 (severely ill) corresponded to PANSS-FSNS scores of 24, 27, 30, and 35. PANSS-FSNS scores improved over time, with higher CGI-S scores generally linked to higher estimated PANSS-FSNS scores throughout the study; at week 26, CGI-S scores of 1 (normal), 2 (borderline ill), 3 (mildly ill), 4 (moderately ill), and 5 (markedly ill) corresponded to PANSS-FSNS scores of 7, 12, 18, 22, and 27 **(**Figure S5A**)**. The by-weekly pattern was similar for CGI-S and PANSS-NSS **(**Figure S5B**)**.

When looking at percentage improvement by week **(**Figure S6**)**, linking CGI-I with the estimated PANSS-FSNS percentage improvement at week 26 showed that a rating of minimally improved (3) corresponded to 32%, much improved (2) corresponded to 52%, and very much improved (1) corresponded to 100%. A time effect was noted, indicating that smaller PANSS-FSNS reductions were needed in earlier weeks than at later weeks for patients to be considered improved according to the CGI–I. The pattern was again similar for CGI-I and PANSS-NSS (Figure S6B). Additional by-weekly analyses are presented in Figure S7, S8.

## Discussion

Linking ratings from a psychometric scale with a clinical global impression can help clinicians interpret clinical trial results in a more meaningful way. We performed post hoc equipercentile linking analyses on data from a clinical trial of cariprazine versus risperidone in patients specifically selected for having stable schizophrenia and PNS. Our goal was to characterize how the PANSS-FSNS and PANSS-NSS corresponded to the CGI in patients treated with cariprazine or risperidone. Since prospective selection of patients with schizophrenia and PNS is rare in clinical trials, this was a rich opportunity for us to explore the clinical meaning of PANSS-derived negative symptom scores in this exclusive patient population. Importantly, safeguards taken to minimize the presence of positive symptoms and other pseudospecific effects suggest that the observed negative symptom improvement was likely related to genuine treatment effect. In general, our results demonstrated that greater improvement on the PANSS-FSNS and PANSS-NSS corresponded to a clinical impression of greater improvement, as measured by the CGI-I, and less severe disease states, as measured by the CGI-S. This is an important consideration for clinical trials, as well as for everyday practice where clinicians often rely on their own judgement to assess and treat their patients.

Previous analyses have assessed linking the CGI to psychometric rating scales in psychiatric patient populations (eg, acute exacerbation of schizophrenia, depression) to determine the clinical meaning of rating scale change [4, 17–21], but links between the PANSS-FSNS/-NSS and CGI have not been previously evaluated. Our analyses showed that a CGI-S score of moderately ill corresponded to a PANSS-FSNS score of 24, suggesting that the PANSS-FSNS baseline cutoff score of ≥24 used in the cariprazine PNS trial adequately identified patients with moderate-to-severe negative symptoms. Linking absolute change from baseline on the CGI-S and the PANSS-FSNS found that a greater number of CGI-S category reductions corresponded to greater PANSS-FSNS improvement. When CGI-S category improvement and PANSS-FSNS percentage change from baseline were linked, greater PANSS-FSNS percentage improvements corresponded to more steps of CGI-S category improvement.

Linking rating scale percentage change from baseline with the CGI-I additionally helps to define a threshold of clinical relevance since specific percentage decreases are often used to define response to treatment in clinical trials. In our analyses, linking PANSS-FSNS percentage change (adjusted by subtracting 7 points to account for the minimum PANSS score) and CGI-I showed that 27% PANSS-FSNS improvement corresponded to a CGI-I rating of minimally improved, while 49% improvement corresponded to a CGI-I rating of much improved. This is of particular interest in the context of interpreting the clinical relevance of treatment response in the cariprazine PNS trial [7], where ≥20% decrease from baseline in PANSS-FSNS score was the a priori definition of response. Our results suggest that this ≥20% cutoff, as well as a more stringent ≥30% threshold used in post hoc analysis, were well chosen to determine a level of change that was clinically relevant to patients in this negative symptom population.

Baseline severity of illness did not have a strong effect on the link between CGI scores and PANSS-based scores, suggesting that clinical changes in this patient population were adequately measured regardless of the severity of illness at baseline. A lack of effect for baseline severity in these analyses is different from what has been seen in previous linking analyses where a clear baseline severity effect was seen for absolute score change and CGI improvement; namely, less severely ill patients needed less absolute score change than more severely ill patients to be rated as having the same CGI-I score [17–19]. Of note, this baseline severity effect was not present in prior analyses when percentage change was considered. In addition, when by-weekly data were considered, a time effect was observed for percentage reduction, suggesting that a somewhat smaller objectively measured PANSS-FSNS percentage change was necessary in early weeks than in later weeks for patients to be considered improved on the subjectively rated CGI-I. This finding may reflect clinicians’ expectations of improvement after a short duration of treatment than at later stages.

Our findings contribute to the ongoing discussion of what constitutes a clinically meaningful response for patients with negative symptoms of schizophrenia. In a previous analysis linking CGI scores with the Scale for the Assessment of Negative Symptoms (SANS) in patients with PNS [20], the relationship between the CGI subscales and the SANS followed a linear trend, with higher SANS scores linked to greater CGI severity and greater percentage improvement linked to more CGI improvement. Furthermore, in a pooled, post hoc linking analysis using data from patients with acute exacerbation of schizophrenia, 20% improvement in PANSS total score corresponded to a CGI-I score between minimal improvement and unchanged at week 6 [4].

These analyses present novel findings describing the links between PANSS-derived negative symptom scales and clinical global impressions in a unique population of patients with schizophrenia and persistent PNS. Since the equipercentile linking method was used to describe the link between the PANSS and CGI, results refer to the observed difference in a score or percentage from baseline to endpoint (i.e., from before treatment to after treatment). This does not allow us to make judgments on what would be considered a meaningful difference between interventions; rather, we can determine what amount of PANSS change is considered clinically relevant after treatment. Data from the cariprazine- and risperidone-treatment arms were pooled to create a more robust dataset, so linking results were not treatment specific.

Although broad interpretation of these findings is limited because data were taken from a single study of cariprazine versus risperidone, our findings are particularly interesting given our prospectively defined negative symptom population. The strict inclusion/exclusion criteria applied in the PNS study, limit the ability to generalize these findings to other patients with schizophrenia. In addition, ceiling effects cannot be ruled out since patients had to be well enough to cooperate with study procedures. Correlation coefficients between these PANSS-derived scales and the CGI scales were statistically significant; however, linking any outcome measure that has strong psychometric properties with a global impression scale may have inherent limitations. Ongoing discussion about whether the PANSS is addressing all aspects of negative symptoms adds to the complexity and possible limitation of using this scale; although newer psychometric scales are available to measure negative symptoms, the PANSS is considered an appropriate measure for use in current clinical trials [6].

Results of our analyses showed that greater improvements on the PANSS-FSNS and -NSS were linked to clinical impressions of greater illness improvement and less severe disease states in patients with schizophrenia and PNS. Given that scientific dispute remains regarding which rating scale most effectively measures symptom changes in patients with schizophrenia and negative symptoms, our data suggest that PANSS-derived scales adequately measure clinical changes in this patient population. Since the PANSS scale is widely used in clinical trials and is well known among psychiatrists, these results may provide direction to help define severity thresholds and levels of change that represent clinically relevant improvement in patients with negative symptoms of schizophrenia.

## Funding and disclosure

The work was supported by funding from Gedeon Richter Plc. All authors met ICMJE authorship criteria. Neither honoraria nor payments were made for authorship. Gedeon Richter was involved in the study design, collection (via contracted clinical investigator sites), analysis, and interpretation of data, and decided to submit for publication. Authors had full access to the study data and complete discretion in the analysis of data and writing of this report. In the last 3 years Stefan Leucht has received honoraria for consulting from LB Pharma, Lundbeck, Otsuka, TEVA, LTS Lohmann, Geodon Richter, Recordati, Boehringer Ingelheim, and for lectures from Janssen, Lilly, Lundbeck, Otsuka, SanofiAventis and Servier. Á. B., I. L., B.S., K.A., E.S., J.H., and G.N. are employees of Gedeon Richter Plc and report personal fees from Gedeon Richter Plc, outside the submitted work. G.N. and I.L. have patents issued for cariprazine; B.S. has a patent pending for cariprazine. W.E. is an employee of Allergan.

## Supplementary information

Figure S1, S2. CGI Versus PANSS-FSNS Percentage Change Without 7 Point PANSS Score Adjustment

Figure S3, S4. Median Split Analyses: Linking CGI-I With PANSS-FSNS and -NSS Changes

Figure S5. By Week: Linking CGI-S With PANSS-FSNS (A) and PANSS-NSS (B) Score (Observed Cases)

Figure S6. By Week: Linking CGI-I With PANSS-FSNS (A) and PANSS-NSS (B) Percentage Change (Observed Cases)

Figure S7. By Week: Linking CGI-I With PANSS-FSNS (A) and PANSS-NSS (B) Change (Observed Cases)

Figure S8. Linking CGI-S Change With PANSS-FSNS and PANSS-NSS Score Change (A, B) and Percent Change (C, D) (Observed Cases)
