# Supplementary material for: Linking PANSS negative symptom scores with the Clinical Global Impressions Scale: understanding negative symptom scores in schizophrenia
Source: Neuropsychopharmacology. 2019 Mar 5;44(9):1589–96. doi: 10.1038/s41386-019-0363-2 (PMC6785000; doi:10.1038/s41386-019-0363-2)

Figure S1, S2. CGI Versus PANSS-FSNS Percentage Change Without 7 Point PANSS Score Adjustment

Figure S1. Linking CGI-I Score With Unadjusted PANSS-FSNS (A) and PANSS-NSS (B) Percentage Change (Pooled Observations, Observed Cases)

A. PANSS-FSNS (unadjusted)

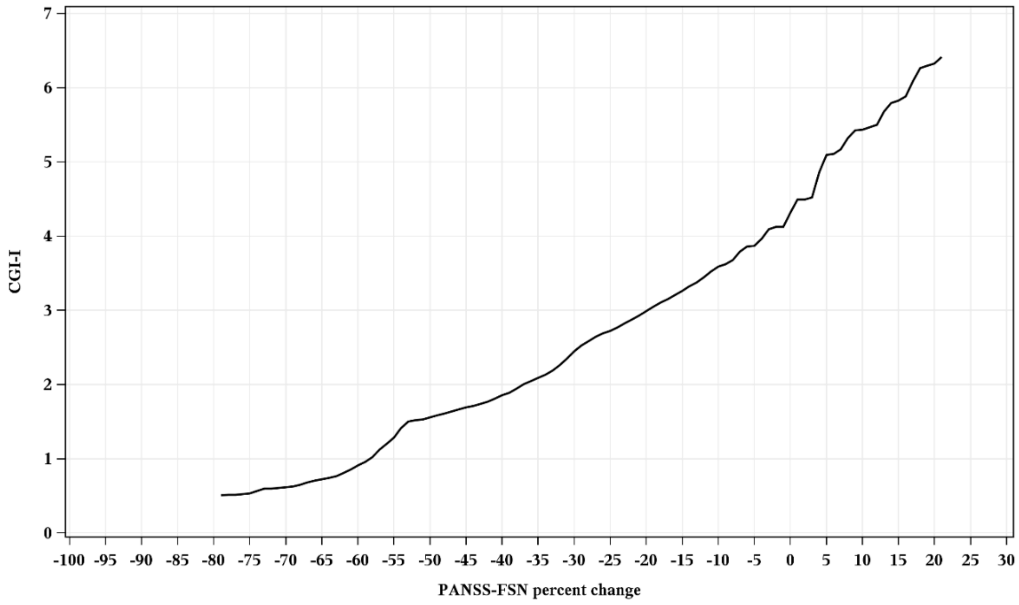

B. PANSS-NSS (unadjusted)

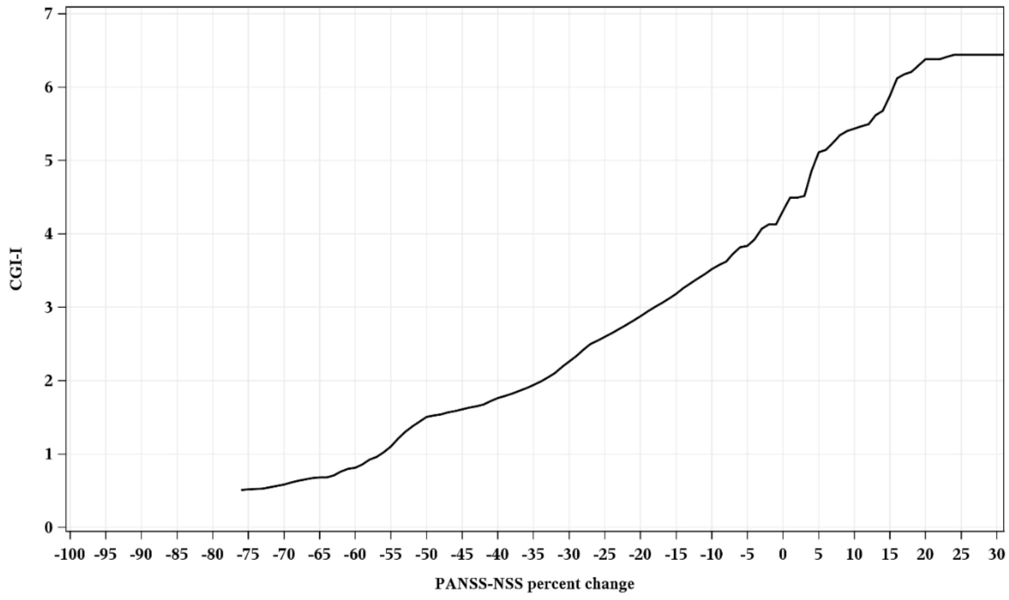

Figure S2. Linking CGI-S Change With Unadjusted PANSS-FSNS (A) and PANSS-NSS (B) Percentage Change (Pooled Observations, Observed Cases)

A. PANSS-FSNS (unadjusted)

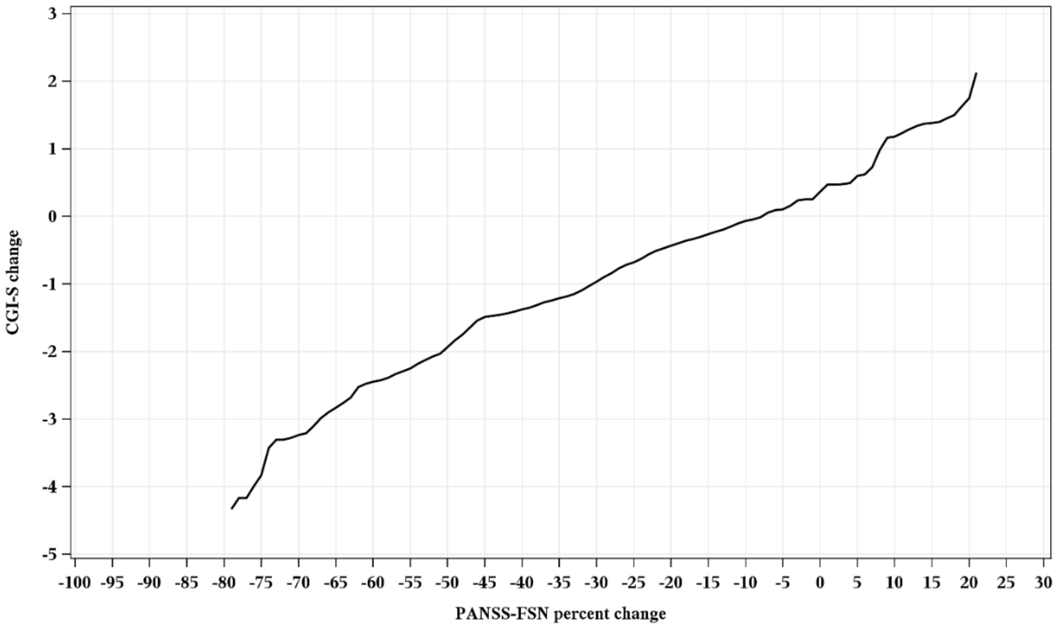

B. PANSS-NSS (unadjusted)

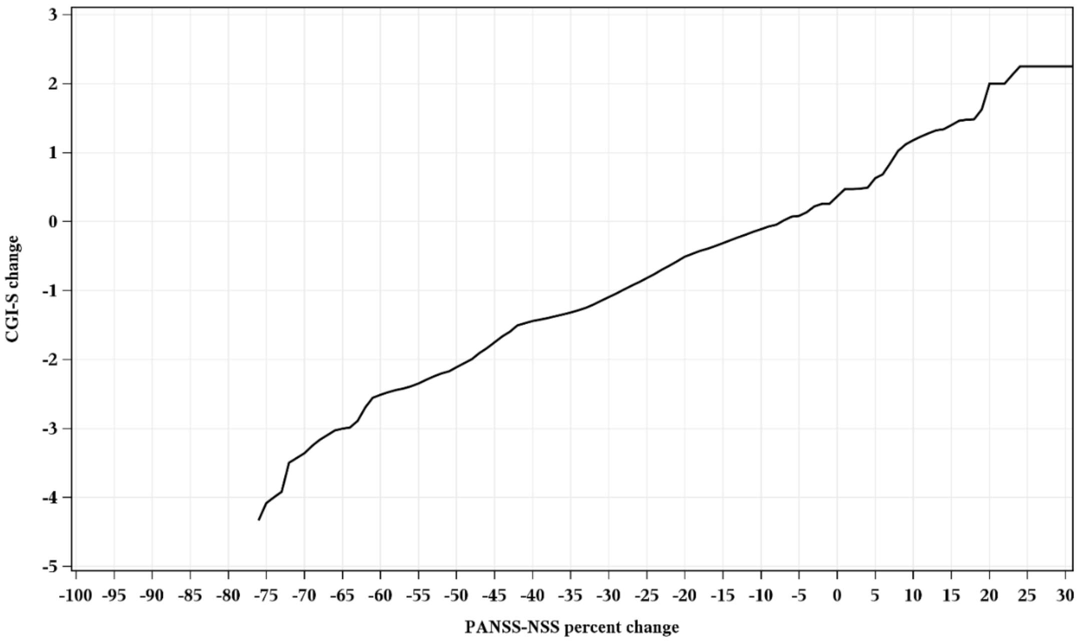

Supplement: Supplementary file 1 — Figure S1, S2. CGI Versus PANSS-FSNS Percentage Change Without 7 Point PANSS Score Adjustment [file 41386_2019_363_MOESM1_ESM.pdf]
