# Supplementary material for: Linking PANSS negative symptom scores with the Clinical Global Impressions Scale: understanding negative symptom scores in schizophrenia
Source: Neuropsychopharmacology. 2019 Mar 5;44(9):1589–96. doi: 10.1038/s41386-019-0363-2 (PMC6785000; doi:10.1038/s41386-019-0363-2)

Figure S3, S4. Median Split Analyses: Linking CGI-I With PANSS-FSNS and -NSS Changes

Figure S3. Median Split: Linking CGI-I With PANSS-FSNS (A) and PANSS-NSS (B) Change (Observed Cases)

A. PANSS-FSNS

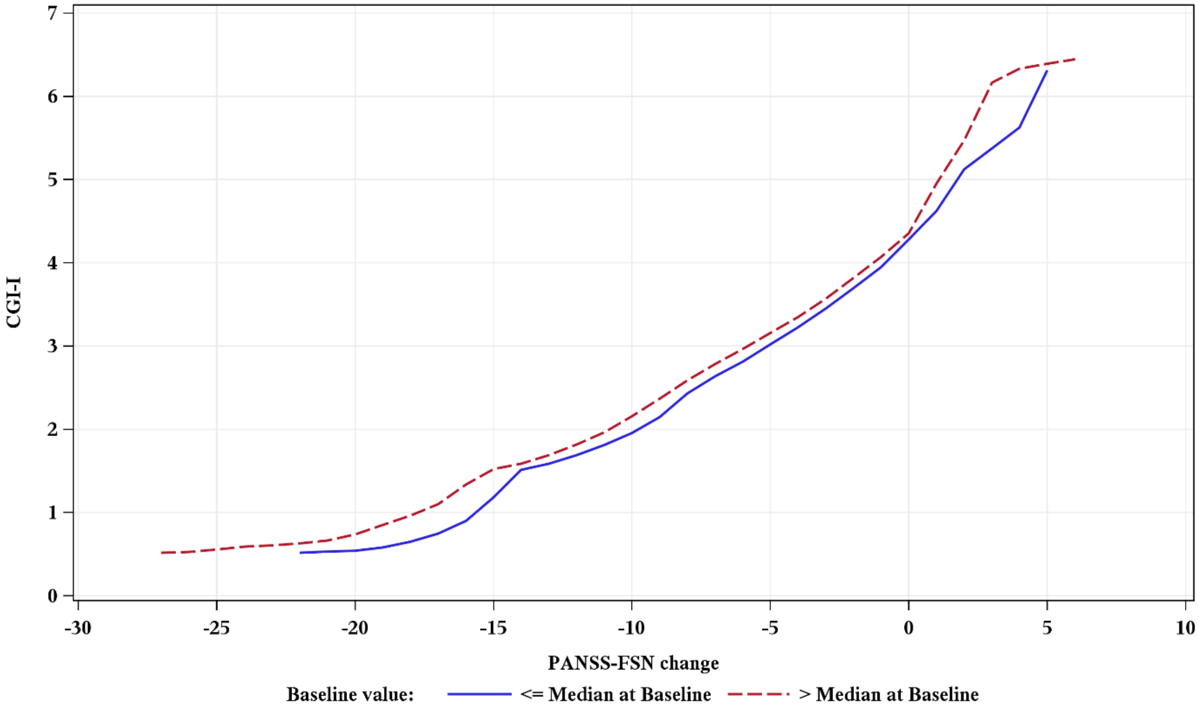

B. PANSS-NSS

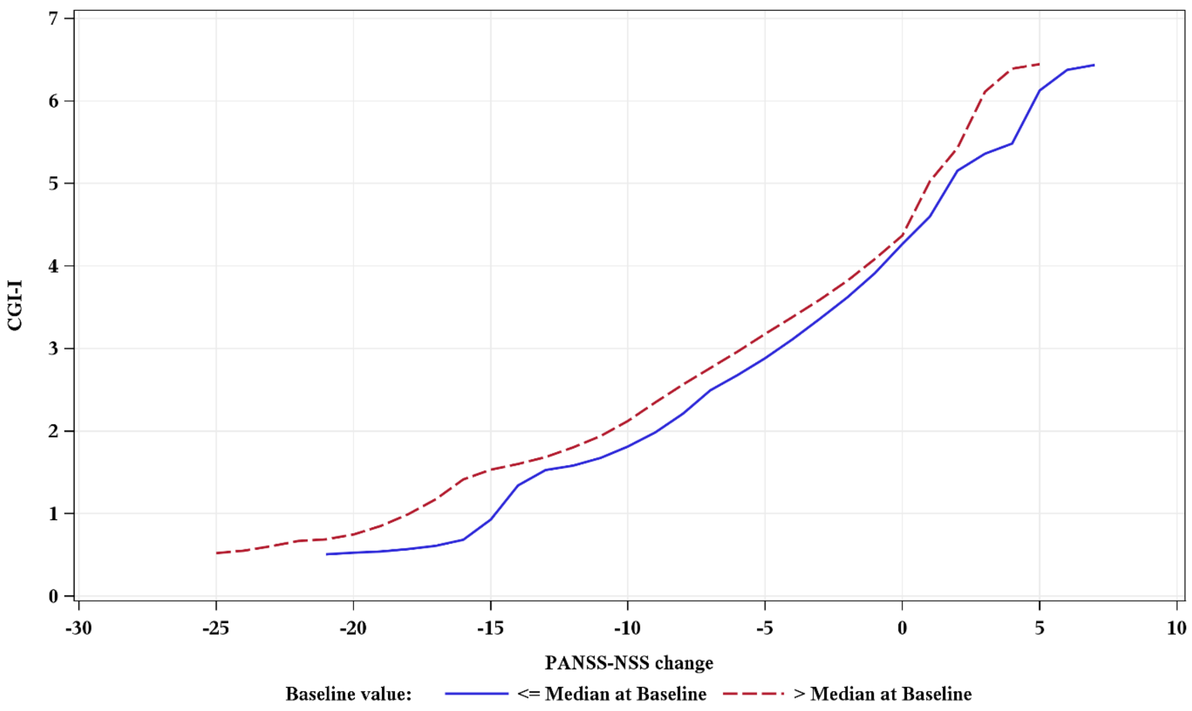

Baseline severity had very little, if any, effect on the correlations between CGI-I score and PANSS-FSNS change (**Figure 3A**) and PANSS-NSS change (**Figure 3B**).

In patients who improved over the course of the study, baseline illness severity had no effect on the link between CGI-I and percentage change on the PANSS-FSNS (**Figure 4A**) and PANSS-NSS (**Figure 4B**).

Figure S4. Median Split: Linking CGI-I With PANSS-FSNS (A) and PANSS-NSS (B) Percentage Change (Observed Cases)

A. PANSS-FSNS

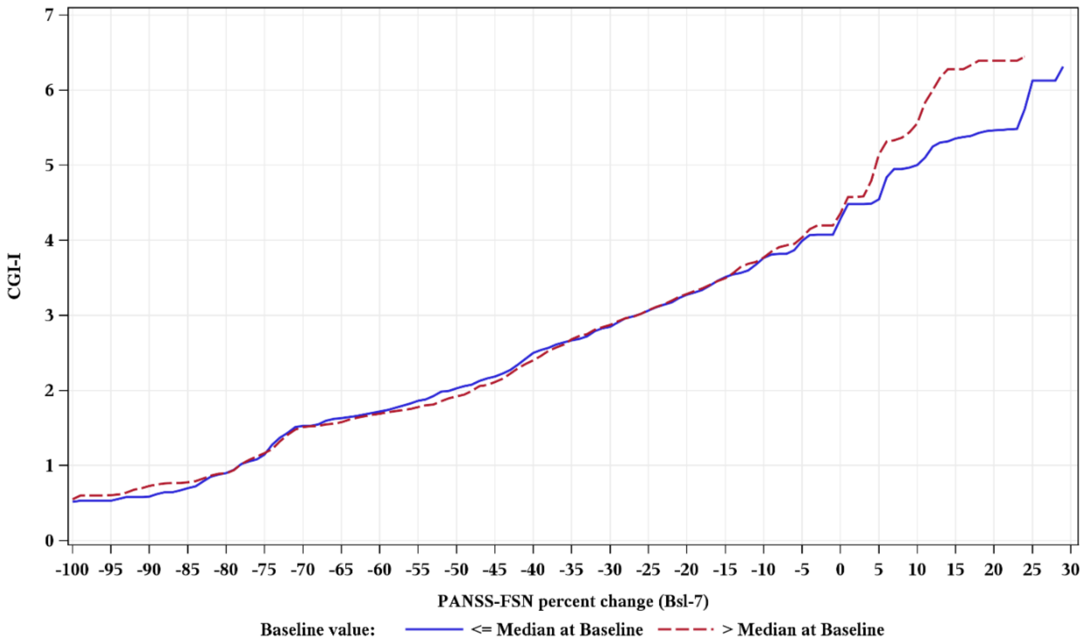

B. PANSS-NSS

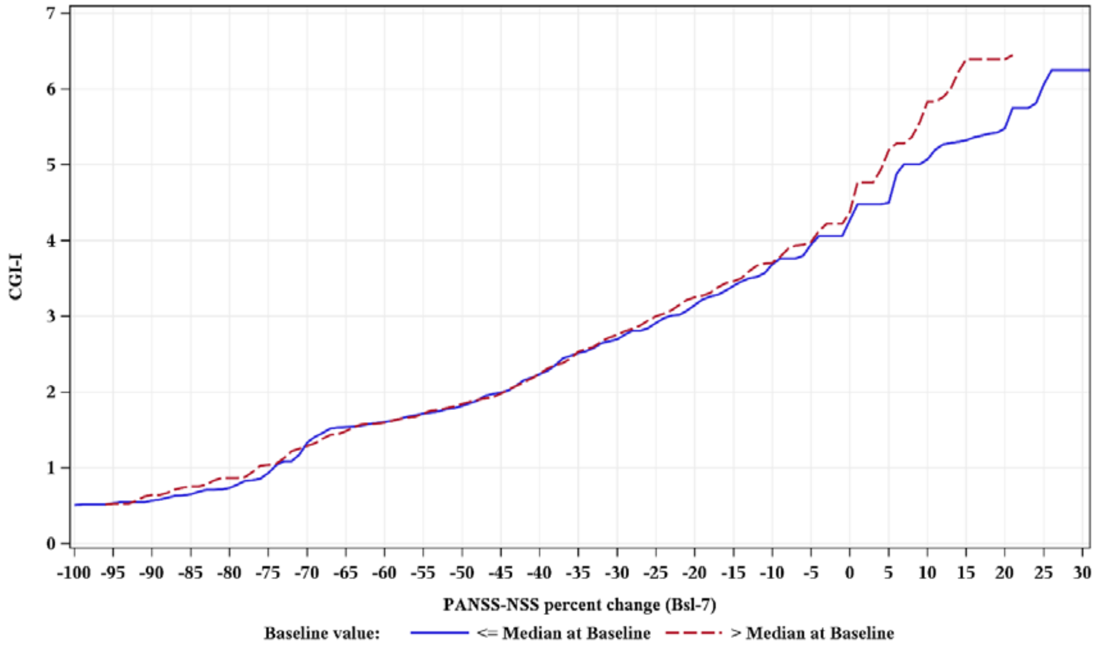

Supplement: Supplementary file 2 — Figure S3, S4. Median Split Analyses: Linking CGI-I With PANSS-FSNS and -NSS Changes [file 41386_2019_363_MOESM2_ESM.pdf]
