# Supplementary material for: Linking PANSS negative symptom scores with the Clinical Global Impressions Scale: understanding negative symptom scores in schizophrenia
Source: Neuropsychopharmacology. 2019 Mar 5;44(9):1589–96. doi: 10.1038/s41386-019-0363-2 (PMC6785000; doi:10.1038/s41386-019-0363-2)

**Supplementary Figure 5. By Week: Linking CGI-S With PANSS-FSNS (A) and PANSS-NSS (B) Score (Observed Cases)**

## A. PANSS-FSNS

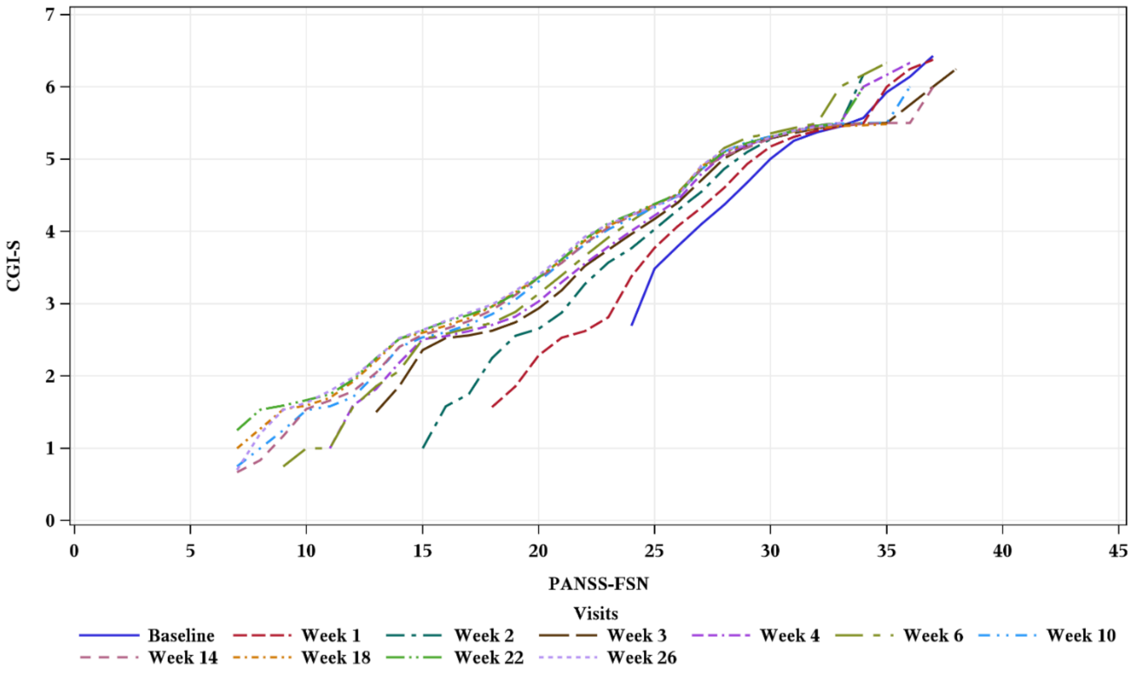


## B. PANSS-NSS

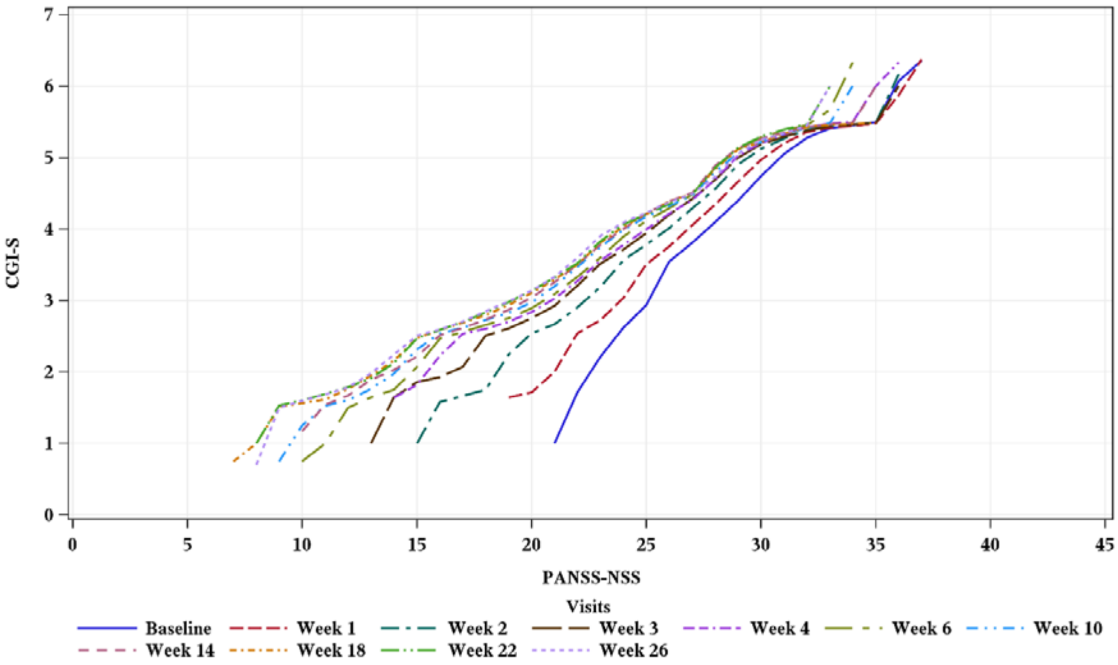

Supplement: Supplementary file 3 — Figure S5. By Week: Linking CGI-S With PANSS-FSNS (A) and PANSS-NSS (B) Score (Observed Cases) [file 41386_2019_363_MOESM3_ESM.pdf]
