# Supplementary material for: Linking PANSS negative symptom scores with the Clinical Global Impressions Scale: understanding negative symptom scores in schizophrenia
Source: Neuropsychopharmacology. 2019 Mar 5;44(9):1589–96. doi: 10.1038/s41386-019-0363-2 (PMC6785000; doi:10.1038/s41386-019-0363-2)

Supplementary Figure 6. By Week: Linking CGI-I With PANSS-FSNS (A) and PANSS-NSS (B) Percentage Change (Observed Cases)

A. PANSS-FSNS

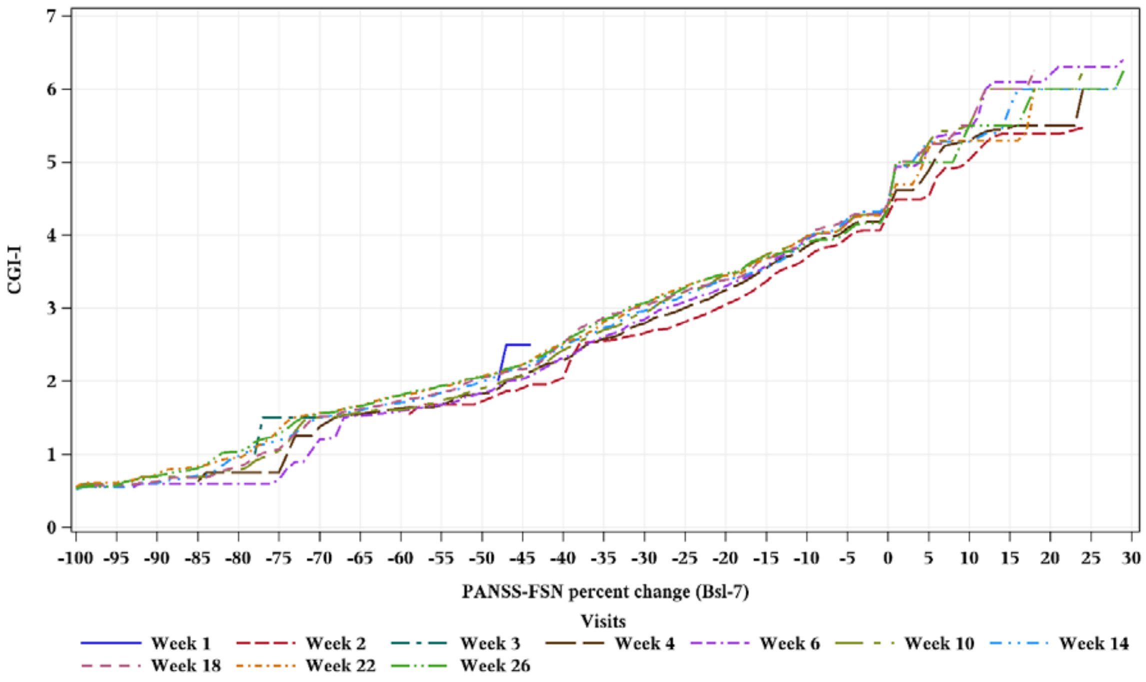

B. PANSS-NSS

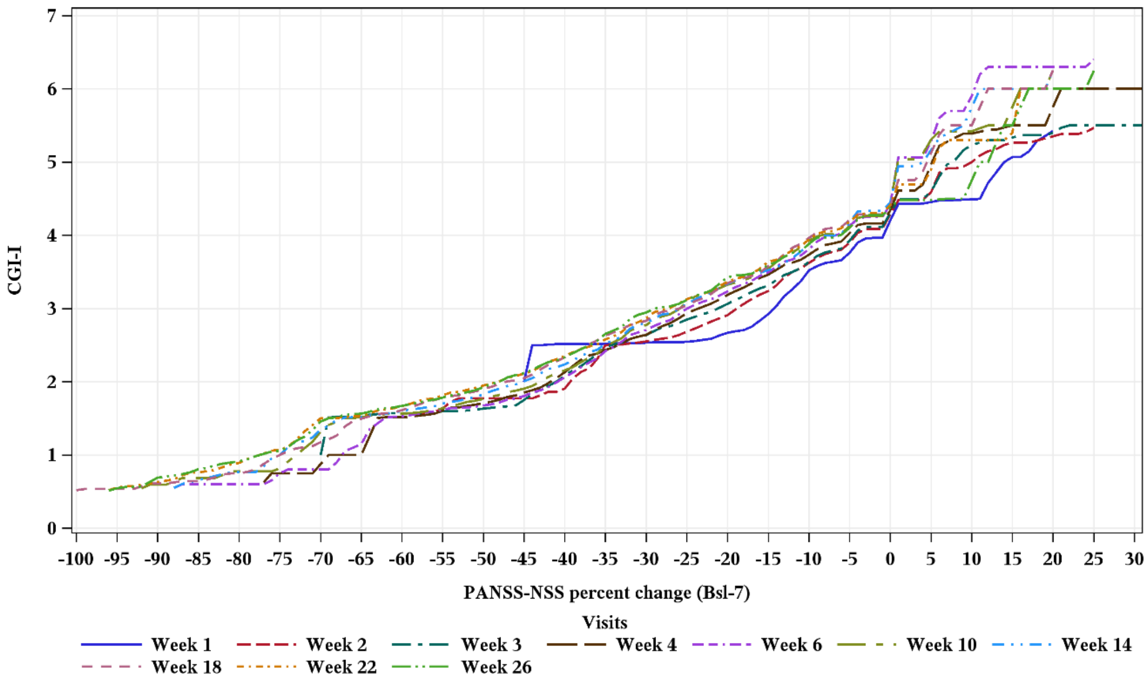

Supplement: Supplementary file 4 — Figure S6. By Week: Linking CGI-I With PANSS-FSNS (A) and PANSS-NSS (B) Percentage Change (Observed Cases) [file 41386_2019_363_MOESM4_ESM.pdf]
