# Supplementary material for: Linking PANSS negative symptom scores with the Clinical Global Impressions Scale: understanding negative symptom scores in schizophrenia
Source: Neuropsychopharmacology. 2019 Mar 5;44(9):1589–96. doi: 10.1038/s41386-019-0363-2 (PMC6785000; doi:10.1038/s41386-019-0363-2)

Supplementary Figure 8. Linking CGI-S Change With PANSS-FSNS and PANSS-NSS Score Change (A, B) and Percent Change (C, D) (Observed Cases)

A. PANSS-FSNS Score Change

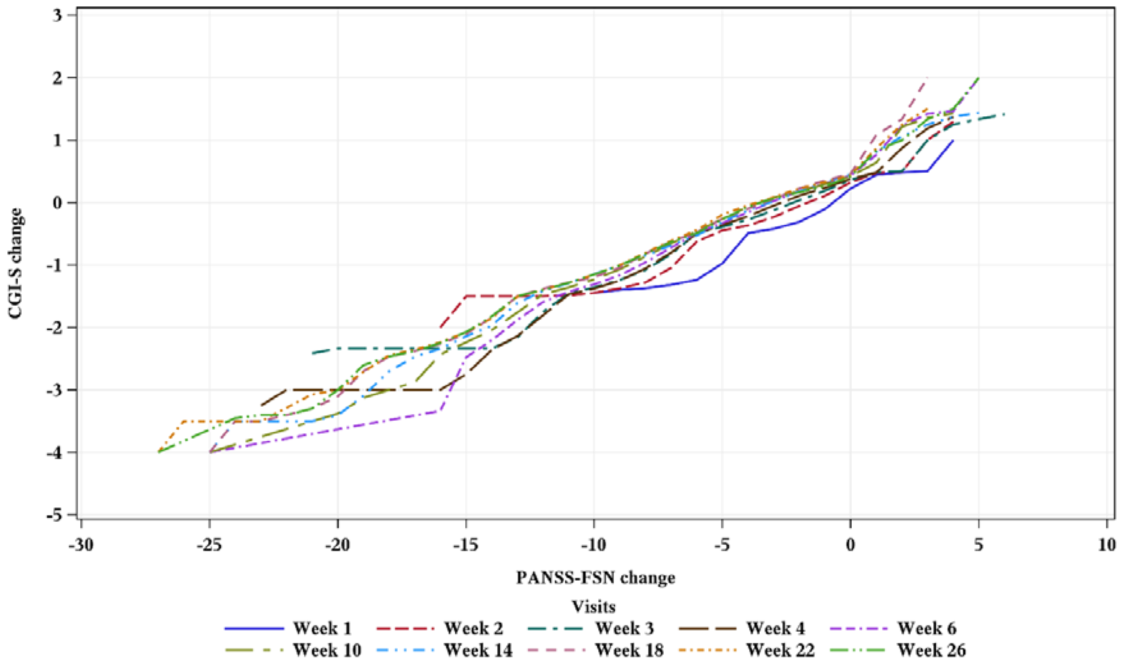

B. PANSS-NSS Score Change

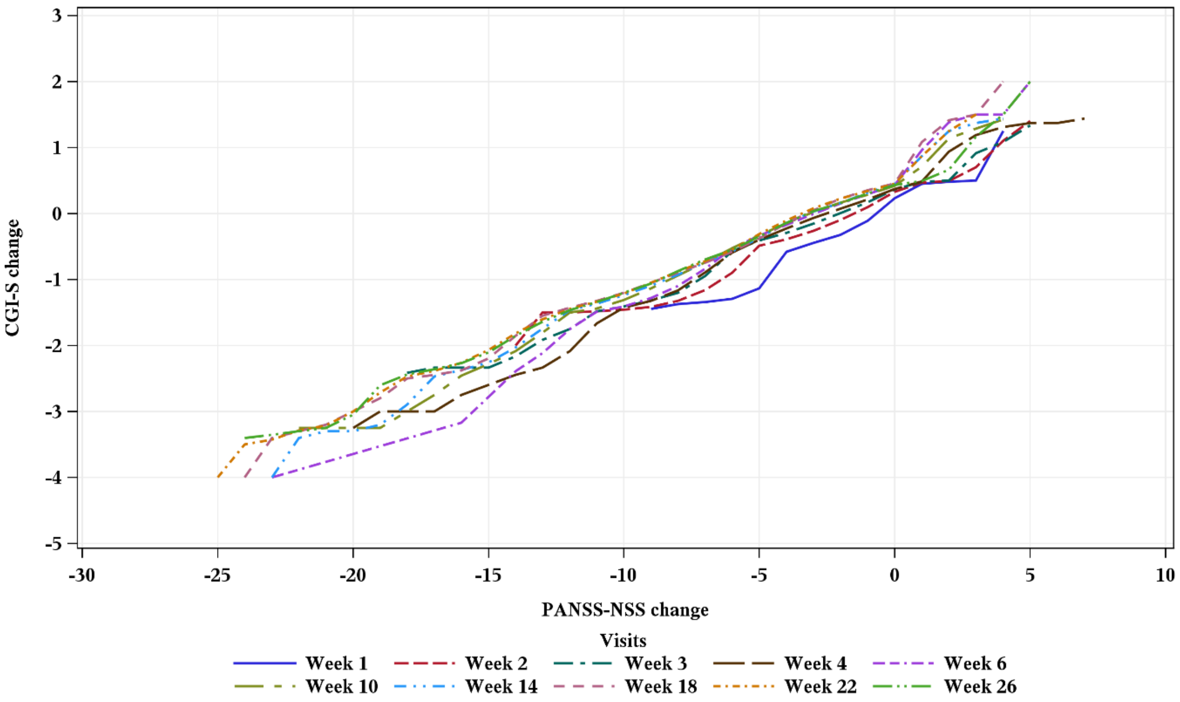

C. PANSS-FSNS Percent Change

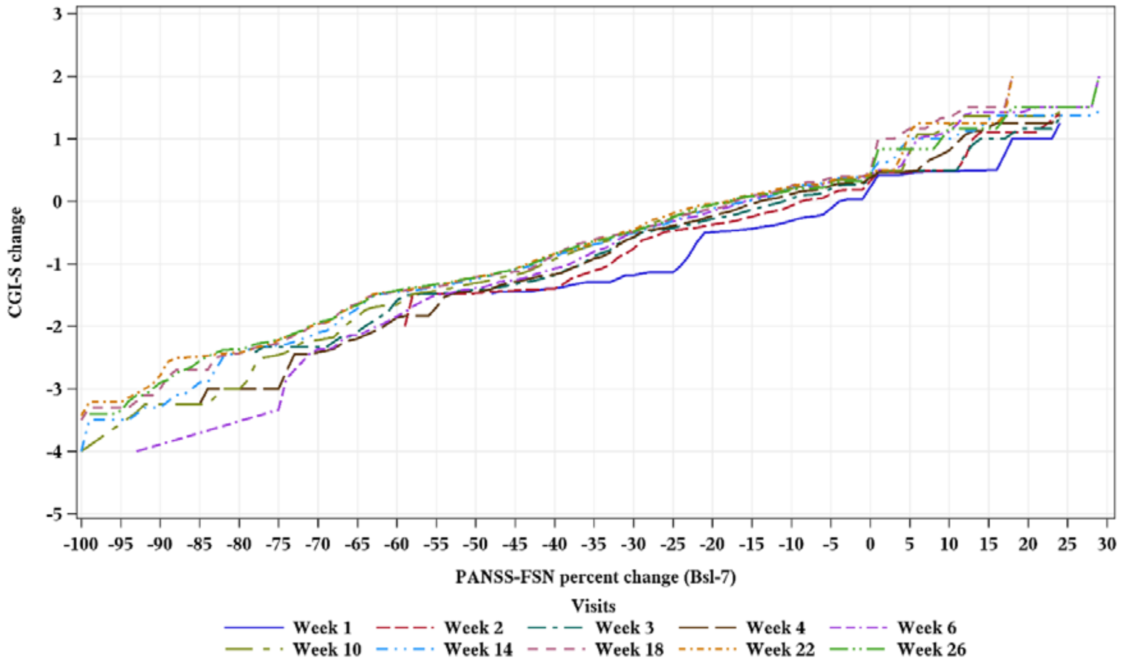

D. PANSS-NSS Percent Change

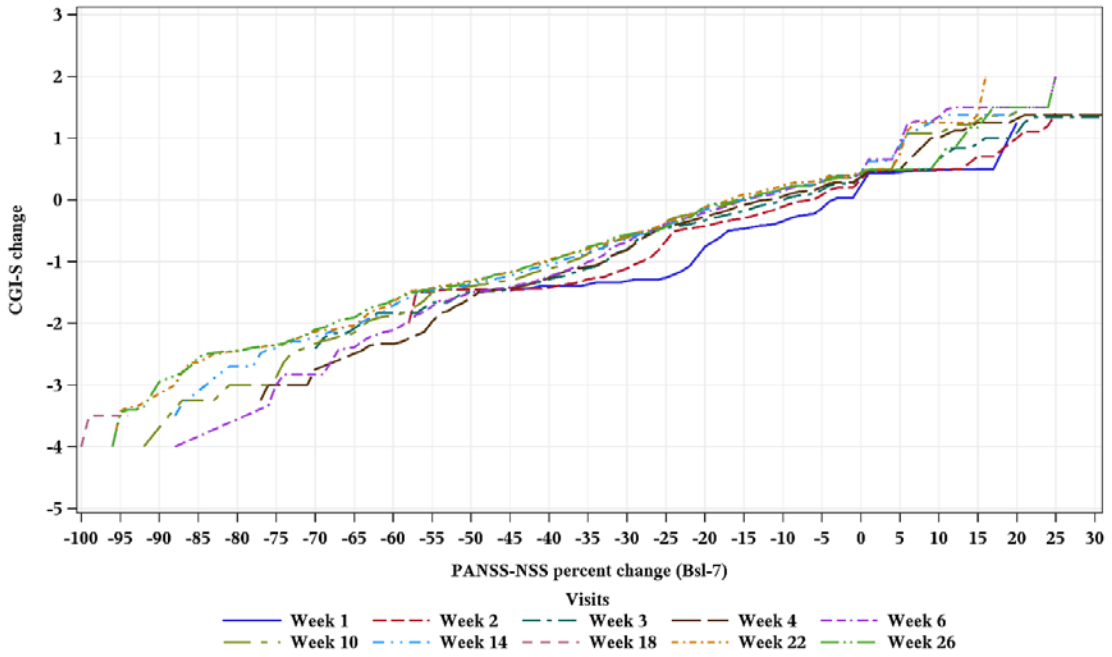

Supplement: Supplementary file 6 — Figure S8. Linking CGI-S Change With PANSS-FSNS and PANSS-NSS Score Change (A, B) and Percent Change (C, D) (Observed Cases) [file 41386_2019_363_MOESM6_ESM.pdf]
